# Case Report: IgG4-RD-related autoimmune pancreatitis combined with monoclonal gammopathy of undetermined significance

**DOI:** 10.3389/fimmu.2025.1729602

**Published:** 2026-01-07

**Authors:** Jinhu Wei, Xiang Chen, Li Chen, Yao Xie, Wanman Li, Jiyuan Liang

**Affiliations:** 1 Department of Laboratory Medicine, Liuzhou Municipal Liutie Central Hospital, Liuzhou, China; 2 Department of Hematology Medicine, Liuzhou Municipal Liutie Central Hospital, Liuzhou, China; 3 Department of Rheumatology and Immunology, The Second Affiliated Hospital of Guangxi University of Science and Technology, Liuzhou, China

**Keywords:** IgG4-related autoimmune pancreatitis, immunofixation electrophoresis, M protein, serum protein electrophoresis, unexplained monoclonal gammopathy of undetermined significance

## Abstract

Immunoglobulin G4-related disease (IgG4-RD) is an immune-mediated chronic fibrotic inflammatory disorder. The coexistence of IgG4-RD with monoclonal gammopathy of undetermined significance (MGUS) is relatively rare, with only a few cases reported in the literature. This article reports a case of a 79-year-old male patient with clinical manifestations of persistent upper abdominal pain of unknown cause and recurrent pleural effusion. Auxiliary examinations indicated positive serum antinuclear antibodies and persistently positive IgG4; three-dimensional imaging reconstruction showed mild pancreatic swelling and the peripancreatic fat planes appeared blurred, which was consistent with the manifestations of pancreatitis. According to the “International Consensus Diagnostic Criteria for Autoimmune Pancreatitis (AIP)”of the diagnostic criteria of the International Pancreatic Disease Association, the patient was diagnosed with IgG4-RD AIP. At the same time, the patient’s serum protein electrophoresis M protein was 2.34 g/L, and immunofixation electrophoresis confirmed it to be IgG-λ-type M protein. Combined with bone marrow smear and biopsy results, it was diagnosed as having MGUS. To our knowledge, this represents a rare case of IgG4-RD AIP concomitant with MGUS, and we provide an in-depth discussion of its clinical management and potential pathogenic association.

## Introduction

IgG4-RD is an immune-mediated fibrotic inflammatory disorder, typically presenting with a slow, insidious or subacute onset. Its core clinical features include significantly elevated serum IgG4 levels and involvement of multiple organs and systems. The characteristic of MGUS is monoclonal proliferation of plasma cells or B cells, which secrete monoclonal immunoglobulins. However, the degree of proliferation has not reached the level that causes damage to the related organs, thus it is defined as a benign, potential precursor state (2). This article reports the diagnosis and treatment process of a patient with IgG4-RD AIP combined with MGUS.

## Case description

A 79-year-old male patient was admitted to the hospital on June 29, 2023 due to “upper abdominal pain for half a day”. The patient reported the onset of intermittent, dull, upper abdominal pain half a day prior to admission, without any obvious trigger. The pain was mild and self-resolving, accompanied by acid reflux, abdominal distension, nausea, and a sensation of a foreign object in the throat; he also had poor appetite, fatigue, and significantly reduced food intake compared to before, and occasionally had coughing. The patient had a history of chronic gastritis and recurrent bilateral foot rashes with itching for many years. On June 25, 2023, he underwent percutaneous coronary intervention (PCI) for coronary artery stenosis. He denied other medical history and food/drink allergy history. Since the onset of the disease, the patient has repeatedly experienced pleural effusion. Despite treatments such as diuresis, thoracic puncture for fluid drainage, and anti-inflammatory therapy, the pleural effusion still recurs, and the upper abdominal pain has not been relieved. On June 19, 2023, the gastroscopy indicated erosive gastritis, and the colonoscopy indicated rectal sigmoid colitis. On July 3, 2023, chest CT showed bilateral pneumonia with effusion. The serum carbohydrate antigen 125 (CA125) was 55.36 U/ml, and the serum carbohydrate antigen 199 (CA199) was 0.01 U/ml.

**Figure 1:**
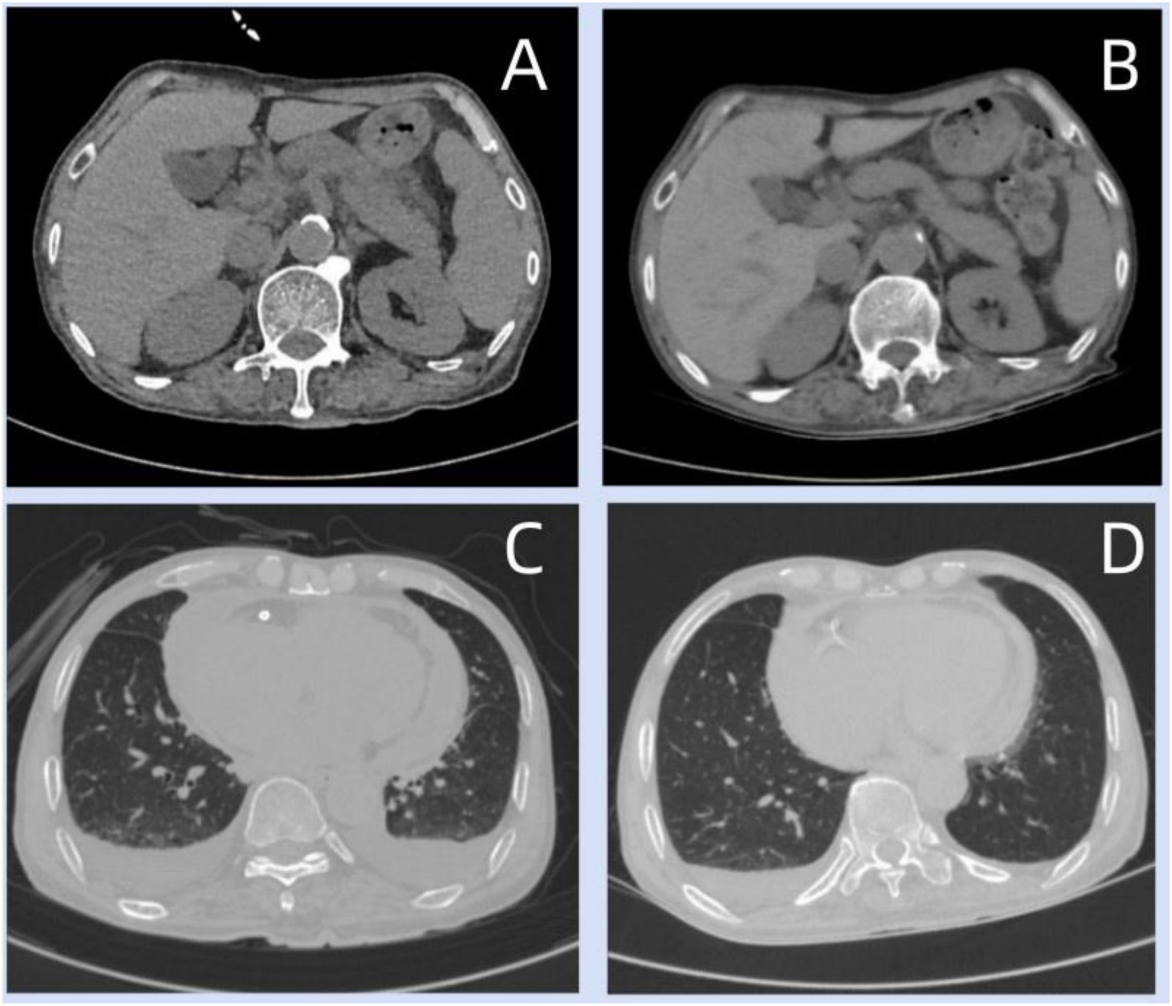
Before hormone treatment, the abdominal CT scan showed mild enlargement of the pancreas and a blurred fat plane around the pancreas **(A)**; after hormone treatment, the CT scan showed that the pancreatic swelling had subsided and the surrounding exudation had largely absorbed. There was a small amount of inflammation in the lower basal segment of the right lung, and there was a small amount of effusion in both bilateral pleural cavities and pericardium **(B)**; the three-dimensional reconstruction of the CT images before hormone treatment showed multiple small nodules in both lungs, with the maximum diameter approximately 0.4 centimeters, located in the posterior segment of the right lung lobe; there were patchy and cord-like high-density shadows in both lungs, with some edges being blurred **(C)**; after hormone treatment, the patchy and cord-like density shadows in both lungs decreased, with some edges becoming blurred, showing a trend of improvement **(D)**.

The auxiliary examination results during hospitalization are as follows: Hemoglobin 96.0 g/L, ESR 106 mm/h, C-reactive protein 43.67 mg/L; The quantitative result of anti-SS-A antibody was 11 U (reference value < 10 U), indicating a mild increase, anti-nuclear antibody is positive; the anti-neutrophil cytoplasmic antibody (ANCA) test showed that myeloperoxidase and PR3 proteinase 3 were negative; no acid-fast bacilli were found in the pleural effusion and sputum smears; no tuberculosis bacilli were cultured from the pleural effusion and sputum;Interleukin-4 is 13.37 pg/ml, Interleukin-6 is 55.14 pg/ml, Interferon-γ is 11.88 pg/ml. Serum globulin 41.1 g/L, IgG 18.51 g/L, λ light chain 2.24 g/L, κ light chain 5.28 g/L; Serum complement C3 0.6 g/L, and serum complement C4 0.05 g/L; Urine free κ light chain 253.21 mg/L, urine free λ light chain 31.46 mg/L. Serum glucose 4.67 mmol/L, 24-hour urine protein 397 mg, urine glucose (+4) and positive fecal occult blood test. No abnormalities were found in blood lipids, liver and kidney functions, electrolytes, myocardial enzymes and troponin. Routine examination of pleural effusion: Rivalta test is positive, total white blood cell count is 2725×10^6^/L, monocytes account for 95%; Pleural effusion biochemistry: lactate dehydrogenase 128 IU/L, adenosine deaminase 10.1 U/L, protein quantification 32.9 g/L, glucose quantification 8.21 mmol/L, chloride 106 mmol/L. Serum lipase 48 U/L, α-amylase 49 U/L. On July 19, 2023, the CA125 of pleural effusion was 2375.60 U/ml. Histopathological examination: Gastric mucosa shows mild chronic inflammation, mild intestinal metaplasia of glandular epithelium, special staining HP (-), PAS expression is normal, immunohistochemistry Ki-67 expression is normal. Pleural effusion pathology did not show cancer cells. Three-dimensional reconstruction of the CT images shows multiple small nodules in both lungs, with the maximum diameter of about 0.4 cm, located in the posterior segment of the right lung lobe; patchy and cord-like high-density shadows in both lungs, with some edges being blurred (**Figure 1C**); the trachea and bronchi are unobstructed, no enlarged lymph nodes are seen in the mediastinum; small patchy and strip-like calcifications are seen on the aorta and coronary arteries; arc-shaped liquid density shadows are seen in the pericardium and left pleural cavity. Three-dimensional reconstruction of the abdomen shows a circular low-density shadow in the S4 segment of the liver, with clear boundaries, no dilation of intrahepatic and extrahepatic bile ducts, no obvious abnormalities in the gallbladder, pancreas, spleen, kidneys and adrenal glands, and a cystic protrusion in the descending part of the duodenum, with a local suspicious thickening of the gastric antrum.

**Figure 2:**
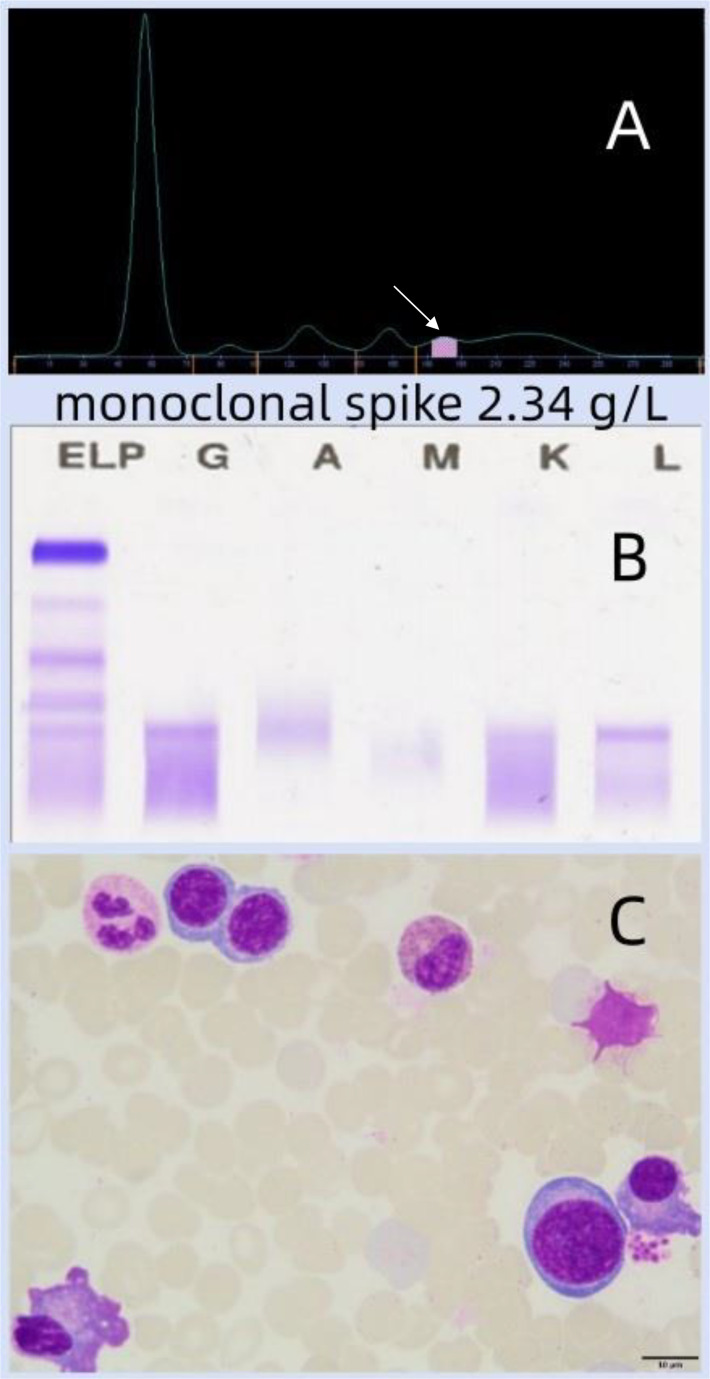
The serum protein electrophoresis (SPEP) revealed a narrow base M protein peak in the γ region (indicated by the arrow), with a quantitative value of 2.34 g/L **(A)**; immunofixation electrophoresis showed IgG-λ type M protein **(B)**. Bone marrow cytology **(C)**: Bone marrow was hyperplastic, with erythroid series significantly hyperplastic, granulocytic series and megakaryocytic series both hyperplastic, platelets scattered or distributed in small clusters, and plasma cell ratio was 4.0%, suggesting monoclonal gammopathy of undetermined significance.

The patient’s serum CA125 level was 55.36 U/ml, and the CA125 level in the pleural fluid was 2375.60 U/ml. Neither imaging nor pathological examinations indicated the presence of a tumor. Therefore, the evidence for a tumor-related disease was insufficient. The serum lipase and α-amylase levels were normal, and no abnormalities were found in the pancreas on imaging, which did not support acute pancreatitis. Laboratory tests (**Figure 2**): A monoclonal band was detected in the γ-globulin region of the serum protein electrophoresis, with a M protein of 2.34 g/L (**Figure 2A**); Immunofixation electrophoresis indicated an IgG-λ type M protein (**Figure 2B**). Bone marrow cytology (**Figure 2C**): The bone marrow was hyperplastic, with significantly active erythroid proliferation, active proliferation of the granulocyte and megakaryocyte systems, scattered or small clusters of platelets, and a plasma cell ratio of 4.0%. This was considered as an unexplained monoclonal gammopathy of unknown significance.

At approximately 17:00 on August 1, 2023, the patient experienced persistent upper abdominal pain that progressively worsened. The pain was more pronounced when lying flat. Accompanying symptoms included profuse sweating all over the body. The patient was considered to have a digestive tract perforation or acute pancreatitis. Immediate blood tests revealed a serum lipase level of 1935 U/L and an α-amylase level of 1012 U/L, both significantly elevated compared to previous results. The quantitative result of anti-SS-A antibody was 11 U (reference value < 10 U), indicating a mild increase, and the anti-nuclear antibody was positive. The serum IgG4 level was as high as 3278.00 μg/mL. Abdominal CT showed a slightly swollen pancreas with the peripancreatic fat planes appeared blurred (**Figure 1A**), Imaging features consistent with IgG4-RD AIP. Given that the anti-SSA antibody level is only slightly above the reference upper limit, and it may be affected by experimental errors, and in the absence of other clinical evidence of Sjögren’s syndrome, its clinical significance is limited. According to the 2019 ACR/EULAR classification criteria for IgG4-related disease, this is not considered an exclusion factor. The patient’s total score reaches 21 points (threshold: ≥20 points), including high serum IgG4 (4 points), imaging showing diffuse pancreatic enlargement and a capsular-like hypointense band (11 points), and hypocomplementemia (6 points), which still supports the diagnosis of IgG4 RD. Considering the significant increase in serum IgG4 and pancreatic imaging changes, it suggests that the patient is in the early or progressive stage of AIP. Further histological biopsy is needed to confirm the infiltration of IgG4^+^ plasma cells and fibrosis characteristics, and an appropriate immunosuppressive treatment plan should be formulated in accordance with the diagnosis and treatment guidelines.

In terms of treatment, Given that the patient has multiple organ involvement and significant serological abnormalities, it indicates a high disease activity level and a threat to the organs. For such critically ill patients, the 2020 international consensus statement recommends the adoption of an aggressive treatment strategy (3). Additionally, existing studies support the application of mycophenolate mofetil in the treatment of IgG4-RD (4). Therefore, we initiated an intensified treatment regimen combining hormones and mycophenolate mofetil. methylprednisolone sodium succinate 40 mg was administered intravenously once daily, combined with mycophenolate mofetil 0.75 g taken orally twice daily for immunosuppressive therapy. Due to the patient’s occurrence of liver function impairment (total bilirubin 79.8 μmol/L, direct bilirubin 43.7 μmol/L, indirect bilirubin 36.1 μmol/L), When selecting alternative medications, we considered cyclophosphamide. According to the 2020 International Consensus Statement on the Management and Treatment of IgG4-related Disease, for refractory IgG4-RD patients who have failed traditional immunosuppressive therapy or are not suitable for using these drugs, cyclophosphamide is considered an important salvage treatment option. Although this patient had not yet experienced the failure of all standard treatments at that time, the severity of the patient’s condition and the risk of progression prompted us to consider this more potent treatment approach. mycophenolate mofetil was discontinued on August 10, 2023, and changed to prednisone tablets 30 mg once daily + oral cyclophosphamide 50 mg once daily. After the treatment, the serum IgG4 gradually decreased. On August 14, 2023, CT scan showed that the pancreatic swelling had subsided, the surrounding exudation had largely absorbed, and there was a small amount of inflammation in the lower basal segment of the right lung; there was also a small amount of effusion in both pleural cavities and pericardium (**Figure 1B**). The patchy and cord-like density shadows in both lungs have decreased, with some edges becoming blurred, indicating a trend of improvement (**Figure 1D**). Subsequently, the patient received oral prednisone tablets at a dose of 10 mg once daily + cyclophosphamide at a dose of 50 mg once daily until January 29, 2024; oral prednisone tablets at a dose of 5 mg once daily were administered from January 30, 2024 to May 29, 2024 for maintenance treatment. On October 24, 2023, the re-examination results were as follows: hemoglobin 118.0 g/L, erythrocyte sedimentation rate 30 mm/h, hypersensitive C-reactive protein 1.97 mg/L, serum lipase 37 U/L, α-amylase 62 U/L, serum IgG4 2061 μg/mL, serum CA125 12.21 U/ml; IgG 13.20 g/L, λ light chain 1.82 g/L, κ light chain 3.62 g/L; Serum complement C3: 1.09 g/L, Serum complement C4: 0.255 g/L. After hormone treatment, the patient’s related clinical symptoms disappeared, and the laboratory and imaging indicators basically returned to normal, which was consistent with the clinical process of IgG4-RD. However, the final diagnosis still requires histopathological confirmation.

## Discussion

IgG4-RD is a chronic and progressive inflammatory fibrotic disease that can affect multiple organs throughout the body, including the pancreas, bile ducts, liver, gastrointestinal tract, prostate, retroperitoneum, skin, and lymph nodes, etc. (5)Although the clinical manifestations of different organs affected vary, its immunological and pathological characteristics share commonalities, mainly including: 1. Elevated serum IgG4 levels; 2. Massive infiltration of lymphocytes and plasma cells in the lesion tissues; 3. Interactions between T cells and B cells mediate inflammatory damage and fibrosis of the organs; 4. Occlusive venous inflammation; 5. Mild to moderate eosinophilic infiltration. Laboratory tests often show significantly elevated serum IgG4 levels. Histopathological examination is the gold standard for diagnosing IgG4-RD, but in clinical practice, it is often limited due to the deep location of the affected areas or difficulty in obtaining tissues (6).

The three-dimensional reconstruction of the patient’s imaging showed mild swelling of the pancreas and the peripancreatic fat planes appeared blurred; the serum IgG4 level was as high as 3278.00 μg/mL; CT suggested bilateral pleural and pericardial effusion. Including the serous cavities, digestive tract and kidneys. Considering the patient’s history of recurrent skin rashes and itching on both feet, the gastroscopy examination revealed erosive gastritis; the colonoscopy examination showed rectal sigmoid colitis; the urine routine test showed glucose (+4) and the fecal occult blood test was positive, which suggested the possibility of multiple system involvement and indicated the potential impact on the exocrine pancreatic organs as well. Although multi-system involvement requires pathological evidence to support it, unfortunately, no pathological examination or immunohistochemical staining results were obtained. However, after hormone treatment, the patient’s related clinical symptoms disappeared, and laboratory and imaging indicators basically returned to normal, supporting the above clinical analysis. According to the “International Consensus Diagnostic Criteria for Autoimmune Pancreatitis (AIP)”of the diagnostic criteria of the International Pancreatic Disease Association (1), this patient meets the PDSHO classification of type 1 AIP: Grade 1 P + any 1/2 grade SOH, supporting the diagnosis of IgG4-RD AIP. Autoimmune diseases may cause an increase in CA125 through inflammatory stimulation of mesothelial cells (such as the pleura and peritoneum), leading to an increase in peripheral blood CA125 (7, 8). In this case, the increase in CA125 may be related to the inflammation of the peritoneum or mesenteric IgG4-RD, and the rapid decrease of CA125 after hormone treatment is consistent with the sensitivity of inflammation to hormones, further supporting the diagnosis of IgG4-RD. Although pulmonary nodules and cord-like shadows are not the most specific manifestations of IgG4-RD (which are more commonly seen as solid masses or interstitial pneumonia), in systemic autoimmune diseases, various non-specific inflammatory responses can occur in the lungs. In this case, the pulmonary lesions significantly improved after hormone treatment, and infection (especially tuberculosis) was excluded. This strongly suggests that it belongs to the same disease spectrum of immune-mediated injury as IgG4-RD AIP. Combined with the good therapeutic effect achieved after 13 days of hormone treatment, this further confirms the diagnosis of IgG4-RD AIP. IgG4-RD AIP often presents with diffuse pancreatic enlargement, and imaging requires differentiation from pancreatic cancer; it can be distinguished through pathological examination and the response to hormone treatment.

MGUS refers to a state where there is a clonal proliferation of plasma cells or B cells, which secrete monoclonal immunoglobulins but do not reach the criteria for malignancy and do not cause organ damage (9). It is regarded as a benign precursor lesion that may progress to lymphoproliferative disorders or multiple myeloma. The prevalence of MGUS increases with age, and it is more common in people over 65 years old. Most patients will not develop obvious diseases, but some may have an increased risk of fractures, renal function impairment, peripheral neuropathy, secondary immune deficiency, and cardiovascular diseases (10–12). There have been reports in the literature of IgG4-related submandibular gland involvement with elevated monoclonal immunoglobulins (13), as well as ocular manifestations of MGUS (14), but no case reports of IgG4-RD AIP combined with MGUS have been retrieved. Although MGUS is usually associated with plasma cell diseases, some studies suggest that it may be a concomitant condition (13, 15). Liang S et al. (14) reported a case of IgG4-related eye disease, with a 50.7% decrease in serum M protein levels after glucocorticoid treatment, confirming that it may be a concomitant condition of IgG4-RD. This patient was diagnosed with IgG4-RD AIP combined with unexplained MGUS, without renal failure, hypercalcemia or anemia (the hemoglobin level increased from 96 g/L to 118.0 g/L after treatment), and no bone destruction or extramedullary tumors were observed. On July 13, 2023, the bone marrow smear showed 4.0% plasma cells. On July 7, 2023, the serum protein electrophoresis M protein was 2.34 g/L, and the subsequent test results on October 11, 2023, January 19, 2024, and July 21, 2025 were 2.24 g/L, 2.19 g/L, and 2.27 g/L, respectively. During the two-year follow-up, there was no significant change in the M protein level, which prompted reflection on the relationship between IgG4-RD and MGUS. It is notable that the increase in serum globulin levels may not be related to the inflammatory response of IgG4-RD, and MGUS is not a concomitant manifestation of IgG4-RD. Although this patient has multiple organ involvement and a significant increase in IgG4, the combination of IgG4-RD and MGUS does not necessarily correlate with more extensive lesions or higher IgG4 levels. Moreover, the progression of IgG4-RD combined with MGUS to multiple myeloma requires long-term monitoring, as clonal evolution may occur occasionally. As Tanaka et al. (16) reported, a case of intracranial IgG4-RD with low-grade active multiple myeloma is extremely rare. In this case, based on the stability of the M protein and no increase in bone marrow plasma cells, the final diagnosis was IgG4-RD combined with MGUS, making the overall disease assessment more complex.

At present, only a few case reports have described the coexistence of IgG4-RD and MGUS. In most cases, M protein levels decreased to some extent after glucocorticoid treatment, but there was no evidence of organ damage or progression to myeloma. A potential pathogenic mechanism linking these conditions is chronic immune stimulation leading to clonal plasma cell expansion. IgG4 RD itself is accompanied by long-term B cell activation and IgG4-positive plasma cell infiltration. Continuous antigen stimulation may provide a “nursery” for clonal plasma cells (17). Inflammatory microenvironments drive the increase of cytokines such as IL-4, IL-10, and TGF-β in IgG4 RD, which can promote plasma cell survival and clonal expansion, thereby generating monoclonal immunoglobulins. However, there are no large sample cohort or prospective studies to evaluate the prevalence and progression risk of MGUS in IgG4 RD patients, and there is still a lack of direct experimental evidence. Although there are theoretical speculations (chronic immune stimulation, inflammatory factors), there is a lack of experimental verification at the molecular level. The research gap is still significant, and systematic studies on large-scale epidemiology, molecular mechanisms, and treatment strategies are urgently needed to clarify the true association between the two and optimize patient management. Drug treatment effects Glucocorticoids can suppress inflammation, and M protein levels decrease in some patients after hormone treatment, suggesting that clonal plasma cells are sensitive to inflammatory signals. Stratify the risk according to the international MGUS risk model (M protein size, immunoglobulin type, free light chain ratio); follow up serum M protein, free light chain, renal function, blood calcium, and hemoglobin every 6–12 months; if M protein increases, bone marrow plasma cells >10%, or organ damage occurs, consider referral to the hematology department for assessment of progression to Monoclonal Gammopathy of Renal Significance/multiple myeloma. For IgG4 RD (including AIP), glucocorticoids are the preferred treatment; if MGUS is present, observe the dynamics of M protein, and consider B cell-targeted drugs if necessary to better control B cell clones.

Both IgG4-RD and MGUS can affect multiple organ systems. Therefore, it is crucial to determine the source of the M protein during the diagnosis process. The diagnosis and treatment process of this case provide valuable references for the clinical management of such diseases.

## Data Availability

The original contributions presented in the study are included in the article/supplementary material. Further inquiries can be directed to the corresponding author.
